# Social determinants for understanding Muslims’ intentions toward seeking mental health help based on the Theory of Planned Behavior

**DOI:** 10.1177/00207640241288193

**Published:** 2024-10-06

**Authors:** Leena Badran, Niveen Rizkalla, Steven P Segal

**Affiliations:** 1 School of Social Welfare, University of California Berkeley, USA; 2 Center for Effective Global Action and The School of Public Health, University of California Berkeley, USA

**Keywords:** Attitudes, behavioral control, intentions, Muslims, qualitative study, seeking mental health help, subjective norms, Theory of Planned Behavior

## Abstract

**Background:**

Many have found that minorities seek help for mental health problems less than the general population. Such findings are surprising considering that minorities experience higher rates of mental health issues compared to the general population.

**Objectives:**

Employing the Theory of Planned Behavior (TPB), this study aimed to explore the intentions of Muslims living in California and Israel pertaining seeking mental health help (SMHH).

**Method and design:**

A qualitative approach involving semi-structured interviews guided by TPB principles was conducted with 78 participants. Thematic analysis was implemented to identify key themes.

**Results:**

Five major themes were identified: 1 – Attitudes: Normalization of SMHH entangled with fear; causes and attributions of mental health disorders; perspectives on treatment. 2 – Subjective norms: Support groups; stigma and social norms. 3 – Perceived behavioral control: Personal and environmental facilitators and barriers. 4 – Intentions: High; conditioned preapproval; and low. 5 – Actual help seeking behavior: Religious figures as first resort; incorporating religious practices, and preference of Muslim therapist. Cultural beliefs, stigma, social support, and religion elements were dominant in the TPB model.

**Conclusions:**

The findings underscored the holistic approach among Muslims toward seeking mental help incorporating medical, psychological, social, and spiritual understanding of the mental health condition. This suggests considering social and communal elements in developing interventions, education, and policy for SMHH among Muslims.

## Introduction

Numerous studies have underscored the inadequate and underutilization of mental health services within ethnic groups globally, a surprising trend given the elevated prevalence of mental health disorders among these populations (Al-Krenawi & Graham, 2011; Awaad et al., 2022; Seeman et al., 2016). Delay in seeking mental health help (SMHH) among minority populations was found to disrupt education, limit job opportunities, increase economic costs, and relate to higher death rates compared to the general population (Niendam et al., 2023). The sooner individuals seek mental health assistance, the better the outcomes and control they can achieve over their mental health condition. Thus, ensuring equitable access to mental healthcare is crucial for enhancing outcomes among individuals from various ethnic backgrounds (Klaufus et al., 2014).

There is an increased attention to understanding help-seeking behaviors for mental health problems due to its effect on long-term prognosis. Timely interventions for mental health problems can alleviate the suffering of affected individuals and help prevent the progression of psychological issues into chronic mental illnesses (Christodoulou & Christodoulou, 2007). Even though early interventions offer significant benefits, studies consistently reveal a gap between the need for specialized mental health services and their actual usage among minority groups (Aloud & Rathur, 2009; Herzig, 2011; Jackson Williams, 2014).

The process of seeking mental health assistance involves multiple stages, encompassing awareness of the issue, decision-making regarding help-seeking, reaching out, and the actual utilization of services (Aloud & Rathur, 2009). However, among Muslim populations, this process is influenced by various obstacles, including individual, familial, social, cultural, and environmental factors (Mohammadifirouzeh et al., 2023). Stigma and shame are recognized as significant factors that impede the utilization of mental health services within Muslim communities (Amri & Bemak, 2013; Loya et al., 2010). Additional studies highlight the influence of social structures and prevailing religion beliefs regarding mental health disorders, contributing to a preference for SMHH from informal sources, such as family, friends, community leaders, or support groups (Hamid & Furnham, 2013). Despite being exposed to individualistic values, Muslim families exhibit unwavering dedication to preserving the family unit and upholding collectivist principles, particularly during times of crisis, when they are expected to provide support.

Adaptive and maladaptive strategies were identified when coping with life stressors. Conceptually, coping is multi-dimensional, containing various aspects such as social structure, social support, and religious considerations (Rana et al., 2014). Coping strategies and perceptions pertaining to physical illness are notably associated with adjustment ramifications (Turkington et al., 2018). Muslim Indonesian nurses who coped with the death of their patients used sharing with colleagues, avoidance, and spiritual practices as coping mechanisms to deal with their grief reactions (Betriana & Kongsuwan, 2020). Zimet (1988) defines social support as an individual’s belief in the presence of support sources such as family, friends, or others. This support stems from relationships with family, spouses, or community members and significant others. Those who perceive obtaining strong social support often feel loved, valued, and connected to their community, which can be especially beneficial during times of crisis. Generally, individuals with high levels of social support experience more positive outcomes, greater self-esteem, and a more optimistic outlook compared to those with lower levels of support. Researchers highlight two key factors in social support: the number of available sources, which refers to the quantity of people who can provide assistance, and the level of satisfaction with these support sources (Sarason et al., 1983). Among Muslims, the concept of collective morality is deeply rooted in Islamic teachings, emphasizing social support and a shared responsibility for the well-being of others (Abideen & Abbas, 2021). Collectivism appears to foster emotional security, closeness, and deep connections with important others (Rizkalla & Rahav, 2016). Staying connected with religious communities for social support was recognized as significant for preserving Muslims’ mental health (Ali et al., 2022; Eltaiba & Harries, 2015; Wang et al., 2020). A quantitative study that examined mental health seeking behaviors among Muslims in the USA found that participants were most likely to turn to their families, engage in self-care, embrace their religion, or choose not to seek support at all (Sabado et al., 2023).

Abiding faith and religious belief are also factors in help-seeking choice leading to first choosing consultation with religious leaders and relying on religious beliefs. Supernatural forces were among the most common factors attributed to the causality and symptoms of mental issues among Muslim communities alongside other beliefs that it is a test from God, a punishment for one’s sins, a consequence of a person’s weak faith, or God’s will, with the conviction that only his will can provide a cure (Alhomaizi et al., 2018). A qualitative study that explored the intersection between cultural, religious, and mental health attitudes among Muslims uncovered a lack of understanding of mental illness within these communities. Respondents also expressed notable levels of perceived and self-stigma related to mental health. The study underscored the influential role of families and religious practices/beliefs in shaping perceptions toward mental illness (Mechammil et al., 2019).

The utilization of health models in comprehending help-seeking is crucial for researchers to pinpoint potential mechanisms for enhancing help-seeking intentions. Among the health models, the Theory of Planned Behavior (TPB; Ajzen, 1985, 1991) has been one of the most widely investigated. The TPB posits that the primary determinants of a behavior are the intentions to engage in that behavior. Formulated by Ajzen (Ajzen, 1985, 1991), TPB underscores three pivotal cognitive components that are crucial for explaining behavioral intentions and subsequent actions – *attitudes, subjective norms*, and *perceived behavioral control*. Attitudes toward a behavior reflect an individual’s personal predisposition to engage in it. Subjective norms encompass an individual’s perception of social pressures, including the approval or disapproval of significant others and their opinions about the individual’s engagement in the behavior. Perceived behavioral control considers the impact of personal abilities and external constraints. TPB posits that individuals are more inclined to perform a behavior if they (1) hold positive attitudes toward it, (2) perceive approval from significant others whose opinions they value, and (3) believe they possess the necessary resources and opportunities to engage in the behavior.

Research has underscored the effectiveness of TPB in deciphering a range of health behaviors (Gupte et al., 2022; Hagger et al., 2007; Higgins & Conner, 2003; Hunter et al., 2003). A few studies have considered intentions to seek help from mental health professionals among general medical practice patients (Jingyuan & Hye Kyung, 2020) and college students (Madeleine et al., 2020). To the best of our knowledge, no study has applied the TPB to consider SMHH by Muslim minorities. The current study incorporates social, cultural, and religious factors within the TPB elements to better understand the general context of seeking mental help among Muslims in Israel and California. Both groups are minorities living in societies where the majority population is non-Muslim. Today, Muslims in California comprise only 1% of its general population (Pew Research Center, 2018), whereas Muslims within Israel are approximately 1 million, about 17% of the total population portion (Pew Research Center, 2009). In both contexts, the majority population is non-Muslim and has adapted social-culture norms that tend to lean toward western and individualistic values (Hametner et al., 2021), which generally normalize seeking professional mental health support when needed. By studying these two groups of Muslims, we wanted to explore the diversity within the Muslim populations and examine whether exposure to the dominant population’s socio-cultural norms may influence the attitudes toward mental health within the minority Muslim communities. This comparison allows us to identify commonalities, shared challenges, and universal trends in help-seeking behaviors across different Muslim groups, despite their distinct geographical origins and socio-cultural backgrounds.

## Methods

### Participants and procedure

About 37 Muslims (47%) in California and 41 Muslims (53%) in Israel participated in the study (*N* = 78). Participants were mainly women (75%), had moderate economic status (70%), identified as married (69%), and held at least a baccalaureate degree (36%). Californian participants originated from North Africa (4, 11%), West Africa (1, 3%), Middle East (10, 27%), South Asia (9, 24%), Southeast Asia (1, 3%), and others who identified as African Americans (1, 3%), white Americans (8, 21%), and European Americans (3, 8%), all residing in northern and southern California. Participants from Israel were all Arab citizens of Israel who lived in the northern, central, and southern regions of the country. The sample included mental health professionals, and non-professionals from the general population. Participants’ ages ranged from 18 to 67 years (*M* = 37, *SD* = 12). Almost two equal groups were identified on the religiosity scale: traditional (43%) and religious (40%). While 32% sought professional mental help, only 15% were diagnosed with mental health issues (Table 1).

**Table 1.** Sample characteristics *N* = 78.

Demographic variables	No.	Percentage (%)
Participant’s group		
Muslims in California	37	47
Muslims in Israel	41	53
Age 37 ± 12		
Min	18	
Max	67	
Gender		
Men	20	25
Women	58	75
Education		
B.A	28	36
M.A	18	23
PhD	5	6
Student	11	14
Other	16	20
Marital status		
Single	16	25
Married	21	69
Divorced	3	4
Separated	1	1
Work in mental health sector		
Yes	20	26
No	56	72
Not sure	1	1
Geographical locations (California)		
Northern CA	29	78
Southern CA	8	22
Geographical locations (Israel)		
Northern	11	26
Center	25	60
Southern	5	13
Religiosity		
Very religious	9	12
Religious	31	40
Traditional	34	43
Secular	4	5
Economic status		
High	10	13
Moderate	55	70
Low	13	17
History of seeking professional mental help		
Yes	12	32
No	24	65
Not sure	1	3
History of psychiatric counselling		
Yes	14	20
No	62	80
Diagnosed with mental health issues^a^		
Yes	12	15
No	66	85

a Diagnosis of mental health issues included schizophrenia, anxiety, OCD, and PTSD.

The researchers (first two authors) approached Muslim community centers, educational institutions, and diverse Muslim professional groups for the recruitment process in both California and Israel. Furthermore, a snowball sampling technique was utilized for additional recruitment with the help of participants. Inclusion criteria were being a Muslim who lived in California or Israel, at least 18 years of age, and fluent in English or Arabic. Recruitment and data gathering took place between March and September 2023 and was ceased following data saturation (Saunders et al., 2018), in which additional interviews would unlikely yield new information.

### Instrument and data collection

The study was approved by the institutional review board of the University of California Berkeley (2022-12-15929). The team of co-authors – two Palestinian Israeli citizens Muslim and Christian, and an American Jew – all of whom have worked on mental illnesses related-topics among minorities. The TPB Guidelines (Ajzen, 2006) include 10 open-ended questions: attitudes (advantages/disadvantages; four questions), subjective norms (significant others’ disapproval/approval; four questions), and behavioral control (two questions). The interviews were semi-structured, approximately 90 min, and conducted by the first author via Zoom in participants’ preferred language (English/Arabic). Consent forms were signed verbally. Prior to conducting the interviews, each participant received a link to Qualtrics self-filling demographics. Interviews were audio-recorded, transcribed and translated.

### Data analysis

We utilized the TPB framework for inductive analysis, complemented by a deductive approach to incorporate essential variables, including cultural and religious factors derived from personal attitudes influencing intentions. The TPB served as the overarching framework for categorizing themes and subthemes. The primary author conducted the translation and transcription of interviews, and in collaboration with the second author, independently coded all transcripts. Then they compared the analysis and codified themes that were retrieved from participants’ responses within the three areas of TPB, utilizing deductive thematic approach. Codes and themes were discussed and developed using Braun and Clarke’s (2006) 6-phase method for qualitative thematic analysis.

## Results

**Figure 1.**
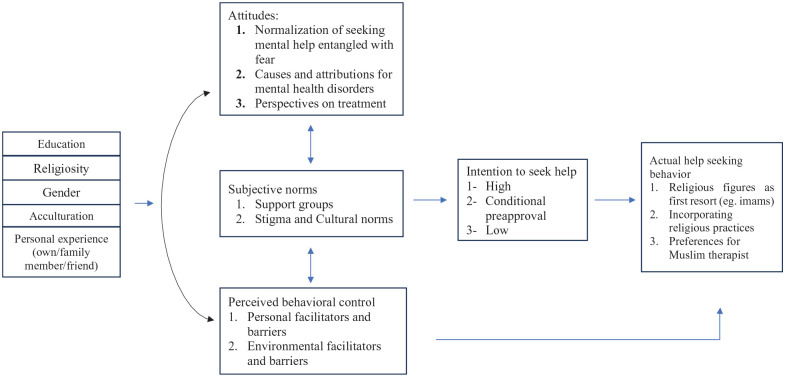
Factors affecting Muslims’ mental help seeking behavior derived from the study results based on TPB (Ajzen, 1991).

Five major themes emerged from the responses: (1) Attitudes, (2) Subjective norms, (3) Perceived behavior control, (4) Intentions, and (5) Actual behavior. Sub-themes are detailed after each major theme (Figure 1).

### Attitudes

#### Normalization of SMHH entangled with fear

All participants declared that SMHH can offer numerous advantages. ‘Who doesn’t need mental health care, right? There’s a lot of stress every day in our lives. . . I’ve personally been through a lot’ (Arab man, 32 years, lives in CA). The benefits encompass improvement in mental health, effective symptom management, valuable guidance and advice, emotional support, as well as enhanced awareness and prevention. SMHH was viewed as a normal process that individuals should adopt and incorporate regularly, regardless of their mental health condition or needs. ‘I often advise people not to wait until they hit rock bottom to consider therapy. It’s beneficial to seek therapy even when you’re feeling content, as it provides an opportunity to discuss your experiences [. . .]’ (Arab woman, 28 years, lives in CA). Some participants explained that normalization of SMHH resulted from the exposure to western values: ‘The emphasis on mental health by non-Muslims around me has led to a growing awareness among Muslims. It is now seen as equally vital as physical health. This shift has normalized and encouraged a proactive approach to mental well-being within the community’ (South Asian woman, 20 years lives in CA).

Stigma emerged as a prominent deterrent and a source of fear for two-thirds of the participants when contemplating mental health care. Fear of being stigmatized was a particularly significant consideration for those in need of care. Despite cultural apprehensions, some participants expressed willingness to seek mental help discreetly. ‘Despite advancements in various aspects of life in America, deeply ingrained beliefs about seeking psychological help persist. While acknowledging the importance of consulting a psychiatrist, cultural and societal pressures create a sense of fear and hesitation’ (North African woman, 45 years, lives in CA).

However, the reluctance to seek mental help among men was linked to socio-cultural expectations, fostering fear of being perceived as mentally soft. As one African American participant expressed, there is a desire to project strength and adhere to traditional male norms, leading to a reluctance to seek assistance, as it may be perceived as a sign of vulnerability.

#### Causes of mental health disorders

With the increasing normalization of SMHH, participants leaned toward attributing mental health problems to biopsychosocial factors, without dismissing spiritual or religious factors. The visibility and severity of symptoms played a pivotal role of participants’ comprehension in determining causality and coping strategies. When symptoms were perceived as manageable and mild, participants attributed them to supernatural powers, and sought resolution through spiritual and religious practices. In contrast, when symptoms were severe, they attributed them to health aspects, and opted for psychiatric sessions as a coping strategy. One participant attributed her husband’s symptoms to supernatural powers, initially seeking assistance from religious figures in the community. However, as the symptoms worsened, she consulted the family physician alongside to relying on religious practices (e.g. prayers, recitation of scriptures) as a coping mechanism. ‘I am a religious woman. I read the Qur’an and believe in God. In the beginning, I was searching for religious causes for mental illness. Is there an ‘[evil] eye’? Is there magic or anything else? What is the cause of his mental illness? Although my sons took their father to a psychiatric, I continue reading Quran and pray’ (Arab woman, 56 years, lives in Israel). On the other hand, some participants expressed the belief that individuals with strong faith would not be vulnerable to mental illness, stating, ‘I believe that people with strong faith are strong enough to avoid becoming mentally ill’ (Arab man, 58 years, lives in CA).

Another participant, who acknowledged the medical origin of a condition, still believes in the existence of supernatural powers as surpassing any other causality: ‘I am a Muslim woman and work in the field. [mental health]. As long as the topics of jinn, magic, and envy are mentioned in the Qur’an, I believe that they exist. As long as it is known that jinn and magic can lead to insanity or signs of mental illness, this means that this exists and the treatment is not medicine, but religion’ (Arab woman, 42 years, lives in Israel).

#### Perspectives on treatment

When asked about intervention options in treating mental disorders or conditions, participants mentioned two types: psychotherapy and medications. Most participants (two-thirds) expressed concerns about medication dependency and potential side effects. ‘I will immediately think about the negative effects of psychiatric medications and how these will affect my daily functioning at home, with my children, with my husband, and at my work. This is the first thing that popped into my head about the side effects caused by taking psychiatric medications’ (South Asian woman, 37 years, lives in CA). However, some participants emphasized that the positive outcomes of psychiatric medication, despite their side effects, outweigh the challenges of not taking the medication and coping with severe symptoms ‘Without medication, this person’s mental health may deteriorate further, even compared to when they are on medication. If symptoms worsen without treatment, controlling them becomes challenging. It’s worth noting that all medications, not just psychiatric ones, can have side effects’ (White American woman, 28 years, lives in CA).

Participants doubted treatment effectiveness, especially when psychiatrists prioritized diagnoses and medications over understanding the patient. Results showed mixed preferences; some favored psychologists for in-depth exploration of root issues, though psychological treatment was perceived as slow and for the long term; whereas others preferred psychiatrists for a quicker, medication-based approach, yet driven by concerns of cost. One woman cited her shattered trust in therapists due to a misdiagnosis of her family member: ‘The [therapist] was making up diseases or diagnosing and saying ‘well we need to do this and that’ and there’s a special mindset in this person - and I say ‘oh [with astonishment] he is just struggling with adjustment here in the US, it is a new country that’s why he’s not adjusted, he’s struggling with. . .’ I have seen some [therapists], sometimes I feel, it could just be an over analysis’ (South Asian woman, 44 years, lives in CA).

### Subjective norms

#### Support groups

Participants referred to their perceptions of whether significant others in their social groups/communities approved or disapproved their quest of SMHH. Results of these subjective norms were mixed. While some received support for SMHH from their close circles (family and friends), others faced opposition from the same circles. Education and previous knowledge on mental health among families promoted such support. Community support was also indicated as promoting SMHH.

I think if I decided to get mental health [care], my family and friends would encourage me [. . .] They are educated [. . .] I would ask the imam as well (West African man, 40 years, lives in CA).

##### Stigma and cultural norms

Despite the efforts of normalization, participants still articulated significant social stigma surrounding mental health issues and SMHH among the Muslim communities.

In my culture and background, seeking help for mental health issues is seen as shameful and can lead to being labeled as “crazy” or “abnormal.” This makes it difficult for me to get the support I need (Arab man, 55 years, lives in CA).

Other participants explained that stigmatization can potentially lead to adverse consequences, which might also contribute to perceptions of weakness or vulnerability:

It leads to stigmatization, potentially impacting future employment prospects, marriage opportunities of my daughters, and social engagement. Since this information becomes part of my medical record, it could have lifelong repercussions, defining and limiting the opportunities available to me (Arab woman, 35 years, lives in Israel).

Another participant described the emotional and behavioral toll of SMHH due to social stigma: ‘Even though I’m educated and work in the mental health field, I’m reluctant to openly admit that I’ve had therapy sessions with a psychiatrist. I even went so far as to purchase psychiatric medication from a pharmacy outside my town to avoid people in my community knowing, since knowing that I have mental issue, this will erase all my achievements’ (Arab woman, 42 years, lives in Israel).

Married women often chose not to disclose their decision to SMHH due to cultural beliefs, stigma on mental health, and fear of shaming. ‘I won’t disclose to my husband’s family, especially at my father-in-law’s house, that I’m seeking psychological treatment due to their strong stigma and the likelihood that they will label me’ (Arab woman, 42 years, lives in CA).

### Perceived behavioral control

Personal and environmental factors that acted as facilitators and barriers to SMHH were detected. Participants’ personal knowledge on mental health stemmed from professional backgrounds, personal experiences, and education, were facilitators in influencing their perspectives on seeking care. ‘I already have knowledge about some organizations [. . .] We know each other (psychologist) for many years for my daughter’ (Turkish woman, 45 years, lives in CA).

Time constraints and financial costs emerged as barriers. The challenge of balancing work- family life, and other responsibilities made it difficult for participants to allocate time for therapy sessions or afford the associated financial costs. ‘As a student, I understand that these sessions will require about one hour per week. However, the challenge for me is finding the time to search for a therapist’ (White American woman, 19 years lives in CA).

Environmental facilitators such as social support, assistance, and technology played vital roles. Participants recounted support from family or friends, whether in accompanying them to mental health settings or recommending therapists as assisting in SMHH. Technology, through remote platforms, eased access to mental health support, reducing transportation efforts and costs while offering scheduling flexibility. ‘Now we live in [a] telemedicine world, so if I can have these conversations virtually that would work within my schedule, versus going to see them and book an appointment and spend like, the whole day wasted to just go there for that’ (Arab man, 32 years, lives in CA).

Mental health care can be expensive, even with insurance. High co-pays, deductibles, or limited coverage created financial barriers to accessing treatment, especially in the US.

Because mental health care can be costly, I prioritize using my resources to address any physical health concerns for myself or my children before considering investing in mental health treatment (North African woman, 38 years, lives in CA).

Conversely, certain geographical areas experienced a shortage of mental health professionals for in-person sessions, including Muslim therapists. This scarcity resulted in extended waiting times or lists, limited provider options, and potential challenges in being socially-culturally understood, thereby restricting access to necessary support.

I live in Southern California, it’s not really a diverse area. So, it’s hard to find that kind of presentation. I think my sister found one a Muslim psychologist that’s far away, one hour and a half to 2 hours from where I live (South Asian woman, 19 years, lives in CA).

### Intentions

Most participants indicated high intentions to seek mental help, emphasizing personal autonomy. In contrast, a subgroup, primarily consisting of religious participants, mentioned that their intentions were contingent on obtaining approval from family members or religious figures before SMHH. ‘I rely on the imam and the Friday sermons for guidance. If mental health becomes a concern in our community, the imam’s endorsement of seeking help could make a significant impact, encouraging those who may not have considered it before including me’ (South Asian man, 47 years, lives in CA).

The third group has low intentions to SMHH and prefers to manage psychological issues independently, believing they don’t require external/professional services. Generally, this group finds itself caught between two cultures: ‘I believe in the strength of my mind to overcome. We understand the [outside] culture but we still obey the rules of the old teachings’ (Arab man, 63 years, lives in Israel).

### Actual behavior of SMHH

Despite the high level of intentions to seek help, most participants turned to religious leaders as a primary step, considering help from psychiatrists or therapists as a last resort. ‘I reached out to an imam or sheikh first, informing them about my consideration of seeking mental health help. After receiving Islamic advice, I then consulted a specialist’ (South Asian woman, 21 years, lives in CA).

Even when recognizing a medical origin for mental issues, some participants believed in the potential effectiveness of spiritual interventions and considered incorporating them into their overall treatment searched. ‘I found talking to sheikhs and imams to be incredibly helpful. They provided guidance, pointing out what I might have missed and offered the words I needed to hear [. . .], it served as a therapeutic experience [. . .] and a valuable source of support during treatment’ (South Asian woman, 19 years, lives in CA). On the other hand, others searched for Muslim therapists:

When my sister needed a therapist for a family issue, we prioritized finding a Muslim psychologist or someone from a similar ethnic background for better understanding of the complexities involved (White American woman, 20 years, lives in CA).

## Discussion

Guided by the TPB, the study illustrates factors affecting intentions toward SMHH among Muslim participants in California and Israel. Our results identified several personal, familial, social, cultural, physical, and environmental elements incorporated with the influence of religion on attitudes, intentions, and behaviors of SMHH. These factors reinforce the utilization of a holistic model to better understand mental health among Muslim populations. More specifically, the holistic approach of the bio-psycho-socio-spiritual model is an ideal framework to explain Muslims’ approach to SMHH.

Our findings indicate that many participants held general positive attitudes toward SMHH, viewing care as significant for their well-being and normalizing the process. Although the field of mental health has been perceived negatively and as a taboo topic among Muslim communities for decades, in recent years, it has also witnessed some shifts toward increased positive and progressive perceptions (Martinez et al., 2020). These positive changes were attributed to exposure to values that consider SMHH as a normal behavior when care is needed, especially when physical symptoms were overtly observed (Yoon et al., 2013). Still, for some participants the act of SMHH involved fear of being stigmatized. While Abo-Rass (2023) claimed that attitudinal barriers were the main determinant of mental health service use, our results revealed that despite the positive attitudes toward SMHH, the social stigma had more influence on participants’ decision-making process. Thus, our findings emphasized that social factors surpassed individualistic attitudes and considerations.

Socio-gender factors were also identified as influencing the attitudes and behaviors of SMHH. Due to social and gendered norms, men were hesitant to SMHH due to fear of being perceived as mentally soft in a society that emphasizes physical and mental strength as masculine traits required in every man (Badran, 2013). Women were also apprehensive in SMHH, attempting to avoid potential stigma (Ciftci et al., 2013) and future prospects (marriage opportunities, work promotions, and parental rights such as child custody), often resulting in being forced to seek help secretly. When the kept secret of SMHH was exposed in front of the Muslim community, the negative association between mental health issues and the societal toll paid by patients, regardless of their gender, contributed to participants’ feelings of being threatened and at risk of being exploited by others. All of which have made stigma a significant barrier to SMHH, as well as an outcome of SMHH. Thus, and despite being acknowledged for years as a socio-cultural barrier in SMHH, more efforts should be directed toward overcoming this challenge.

The two groups of participants in our study enjoyed diversified demographic backgrounds, with the group from Israel being more homogenic (as Arabs citizens of Israel), whereas the Californian group being heterogenic, migrants originated from multiple locations around the world. We expected that such diversification would yield differences in the perceptions and attitudes toward SMHH and coping with life stressors. A major difference between the two groups was observed pertaining to the stigma and cultural norms’ sub-theme. Participants from Israel emphasized that stigma was a greater concern when SMHH compared to Muslim Americans, who reported reaching out to family for support as their top ranked option (Sabado et al., 2023) and overcoming social stigma and cultural mistrust (Amri & Bemak, 2013). One possible explanation is that Muslim Americans often live at a greater distance from their relatives, which can provide a sense of liberation from the potential consequences of exposure. In contrast, Muslims living in Israel are in physical proximity to their nuclear and extended families and relatives, making the process of SMHH more exposed and stressful due to cultural considerations and the heightened presence of social stigma.

Religious beliefs played a crucial role throughout the findings in dictating the causes, intentions, and actual behavioral elements of SMHH in the model among the participants. Individual cultural interpretations of mental conditions can significantly impact individuals’ decision of SMHH and potentially affect the course of their illness (Islam et al., 2015). Similar to other studies (Hamid & Furnham, 2013), participants acknowledged mental health issues as medical conditions. However, some still relied on cultural or religious attributions to explain the causes of mental health issues, which were at times based on the severity of symptoms. ‘Manageable’ symptoms were often attributed to supernatural forces, while severe symptoms like hearing voices or suicidal thoughts were more readily accepted as psychiatric matters. In another study, participants embraced spiritual and biological attributions for mental illnesses, alongside a strong belief in the effectiveness of traditional/cultural treatments and the recitation of Qur’an as viable psychological treatment methods (Bairami et al., 2021). Attribution of mental health conditions to religious explanations still holds as a significant cause for Muslims despite their simultaneous acknowledgment of medical reasons.

Muslims who solely attributed mental health issues to cultural or spiritual causes, resisted medical treatment, leading to potential delays or dropouts from interventions (Lim et al., 2015). As a result, Muslims typically opt to initially seek help from religious leaders to avoid these inconveniences and complexities, which makes religious beliefs one of the preferred coping strategies. Receiving reassurance from community religious leaders about the compatibility of seeking medical help can contribute to increased acceptance and utilization of formal mental health services. Furthermore, Muslims prefer to rely on religious beliefs, religious practices (e.g. prayers, recitation of scriptures), or participate in faith-based organizations in an attempt to manage the adversities caused by the mental condition. A longitudinal study found that religion as a coping mechanism has a strong association with social support though it was not restricted to it. Additionally, it was reported that religion contributed to the reduction of psychological distress in the face of stressful life events (García et al., 2017).

Such a religious coping mechanism was found common among people with psychotic disorders in the United States (Tepper et al., 2001). This might partially explain why most of the participants in our study preferred having a Muslim practitioner who shared with them similar socio-cultural background and religion. This level of compatibility could have been sought to enhance the therapeutic alliance, the sense of being understood and connected to the therapist, or to simply eliminate the need for explanations about socio-cultural and personal traditions, norms, and values. All of which would have ensured an increased likelihood of receiving high quality of treatment and culturally appropriate interventions that do not conflict with Islamic religious beliefs.

It is essential to recognize that the Muslim population is not a monolithic group, but rather a mosaic of diverse societies, cultures, traditions, and experiences. Muslims come from various racial, ethnic, and national backgrounds, each bringing unique religious and spiritual practices, as well as diversified countries of origin and migration histories, all of which contribute to the expressions of distress. As such, any discussion on mental health within these communities must account for these diversities. Muslim minorities living in Western societies, might face challenges related to acculturation, socio-cultural adaptation, navigating individual religious identities while balancing between their sense of personal uniqueness and the desire for cultural belonging (Wang et al., 2020). One study found that among various groups in the USA, Muslims who adhered more strongly to Islamic values were less likely to seek mental health treatment. Additionally, Muslims who were more acculturated to American values were more likely to seek mental health services compared to those who were less acculturated. The top-ranked sources of support among Muslims who faced mental health issues included family, self-care, religion (e.g. prayer), or not seeking support at all (Sabado et al., 2023).

Diversity also exists within Muslim groups from the same ethnic background and geographical place of origin. Variables such as the level of religiosity and acculturation and demographic variables significantly influence attitudes toward SMHH. In a quantitative study conducted among Muslim American participants, the sample comprised 240 Arabs, 119 South Asians, and 44 African American Muslims. Fifty-six respondents identified as ‘Other,’ primarily Turkish and European American. The study found that African Americans aged 18 to 44 years are 6.9 times more likely to have negative attitudes toward help-seeking compared to those aged 45 years and older. Additionally, men of Arab origin are 2.0 times more likely than women of Arab origin to have negative attitudes toward help-seeking, while men of South Asian origin are 2.7 times more likely than women of South Asian origin to exhibit similar attitudes. The study also found that Arabs are less likely to use counseling services compared to African Americans (Khan, 2006). By acknowledging the varied socio-cultural idioms and expressions of distress and religiosity among Muslims, we can better understand how to address their mental health needs in a more nuanced and effective manner. This approach will ensure that interventions are culturally sensitive and responsive to the broad spectrum of experiences among the Muslim populations.

Another component of the holistic model, which participants emphasized was the significance of family and social support systems in three elements of the TPB model: subjective norms, perceived behavior control, and actual behavior. Obtaining approval from family and friends for SMHH was crucial. This highlights that, despite exposure to individualistic values, Muslim families maintained a strong commitment to preserving the family unit and upholding collectivist values. Collectivism entails many familial and social support systems that act as protective factors and positive coping mechanisms when individuals, especially the ones originated from the Middle East, face challenges (Rizkalla, 2022). Findings from other studies indicated that education (Ayazi et al., 2014) and previous experiences (Soliman et al., 2016) with mental health within the family were generally identified as promoting SMHH. Such elements were realized by participants as facilitators that eased SMHH. Additionally, imams’ approval and encouragement of SMHH was perceived as part of the community social support system that promoted care.

When examining mental health support among Muslims, it is crucial to recognize how community-based and religious support systems can function as culturally appropriate and effective alternatives to formal mental health services. Social Support Theory emphasizes the vital role of strong social connections in providing emotional and practical assistance when needed (Vaux, 1988). Social support can act as a buffer, providing protective effects in the face of psychological stress (Alloway & Bebbington, 1987). For Muslims, families, friends, religious leaders, and community members often form the foundation of these networks, offering support and provide comfort that aligns with social-cultural norms and religious values. Muslims typically uphold a collectivist approach to life, prioritizing harmony in relationships and being highly responsive to the needs of others. They are often willing to put the interests of their family, community, or tribe above their own individual needs. Individuals who trust and value their social networks are more likely to seek help from these communal sources, and due to collectivist cultural norms, they receive significant support. This culturally embedded assistance may not always be recognized as ‘help-seeking’ in traditional research, but it can lead to positive outcomes by addressing crisis and distress in ways that do not pathologize the seekers. Therefore, it is important to consider these culturally congruent approaches when assessing mental health support in Muslim communities, as they reflect a broader understanding of care that extends beyond conventional clinical frameworks.

## Implications

Theoretically, utilizing the TPB model provides a framework on SMHH among Muslims that can be applied to other Muslim groups across various regions. Practically, our findings indicated that attitudes, intentions, and behaviors of SMHH have been impacted by various personal, familial, social, cultural, physical, and environmental elements, as well as religion and religious beliefs. Such multidimensionality emphasizes the necessity of utilizing a holistic model when addressing mental health and SMHH among Muslim populations. Physicians can benefit from this holistic model in initially addressing the physical and somatic symptoms of patients, and gradually after trust is established, to refer them to psychiatrists or psychologists who can delve into deeper levels of interventions, as well as overcome some of the fears from social stigmatization.

One of the most significant factors that participants voiced as strongly encouraging help-seeking was gaining support from their social groups. Practitioners who work with the Muslim community may design therapeutic interventions involving family members, religious leaders, and other community figures, so that incorporating them into the decision-making process and outreach can be perceived as supportive and thus enhance the utilization of mental health support services within the Muslim communities. For policy makers, it calls for work on raising awareness and developing more socio-cultural and religiously adapted and accessible mental health services tailored to Muslims.

For Muslims, social and religious beliefs and values permeate all aspects of life. Religion is viewed as a way of life and living that encompasses various areas including culinary, attiring, social and economic matters, and much more. Therefore, treatment methods and interventions should take into account these religious considerations as they influence decision-making processes of clients and the prospect of their recovery.

From a research perspective, this study paves the way for future investigations into the impact of religiosity among Muslims on SMHH. Further studies should explore the distinctions between generations of Muslims, as the positive shift noted on perceptions toward mental health can stem from different directions that could be utilized for the healing and interventions purposes of beneficiaries.

## Limitations

Although the sample included a variety of racial and ethnic backgrounds, it is important to consider additional factors when addressing the issue of SMHH among Muslims. Muslim groups in California represent a diverse range of socio-cultural norms, even though they share a common religion. This diversity presents challenges when attempting to generalize findings to the broader Muslim population in California. Additionally, our study participants are drawn from specific regions within California and Israel, which may limit the applicability of the findings. Muslims in other areas of these regions or countries may have different experiences or attitudes due to varying social, cultural, or economic factors. The Muslim population in Israel is also diverse, including groups such as Bedouins, who may have unique experiences with SMHH that differ from other Muslim groups. The distinct cultural and contextual differences between the groups in California and Israel, influenced by their unique social and political environments, add further complexity and limit the generalizing of the findings. Other factors that may influence attitudes toward SMHH, but were not examined in our study, include age and level of religiosity. Both of these factors have been identified in previous research as significant contributors to SMHH. Their exclusion in our study represents a limitation, as these variables could further contextualize the mental health attitudes observed among the participants.
